# NCS-1 Deficiency Is Associated With Obesity and Diabetes Type 2 in Mice

**DOI:** 10.3389/fnmol.2019.00078

**Published:** 2019-04-03

**Authors:** Olga Ratai, Joanna Hermainski, Keerthana Ravichandran, Olaf Pongs

**Affiliations:** Center for Integrative Physiology and Molecular Medicine (CIPPM), Institute for Cellular Neurophysiology, University of the Saarland, Homburg, Germany

**Keywords:** neuronal calcium sensor-1, obesity, adipocyte, insulin receptor, insulin resistance, depression

## Abstract

Neuronal calcium sensor-1 (NCS-1) knockout (KO) in mice (NCS-1^−/−^ mice) evokes behavioral phenotypes ranging from learning deficits to avolition and depressive-like behaviors. Here, we showed that with the onset of adulthood NCS-1^−/−^ mice gain considerable weight. Adult NCS-1^−/−^ mice are obese, especially when fed a high-fat diet (HFD), are hyperglycemic and hyperinsulinemic and thus develop a diabetes type 2 phenotype. In comparison to wild type (WT) NCS-1^−/−^ mice display a significant increase in adipose tissue mass. NCS-1^−/−^ adipocytes produce insufficient serum concentrations of resistin and adiponectin. In contrast to WT littermates, adipocytes of NCS-1^−/−^ mice are incapable of up-regulating insulin receptor (IR) concentration in response to HFD. Thus, HFD-fed NCS-1^−/−^ mice exhibit in comparison to WT littermates a significantly reduced IR expression, which may explain the pronounced insulin resistance observed especially with HFD-fed NCS-1^−/−^ mice. We observed a direct correlation between NCS-1 and IR concentrations in the adipocyte membrane and that NCS-1 can be co-immunoprecipitated with IR indicating a direct interplay between NCS-1 and IR. We propose that NCS-1 plays an important role in adipocyte function and that NCS-1 deficiency gives rise to obesity and diabetes type 2 in adult mice. Given the association of altered NCS-1 expression with behaviorial abnormalities, NCS-1^−/−^ mice may offer an interesting perspective for studying in a mouse model a potential genetic link between some psychiatric disorders and the risk of being obese.

## Introduction

Sensing and regulating intracellular levels of calcium are essential for proper function of neuronal and non-neuronal cells. This includes important cellular processes such as the release of neurotransmitters and hormones, vesicular traffic, exo- and endocytosis. Since its discovery in *Drosophila melanogaster* (Pongs et al., 1993), neuronal calcium sensor-1 (NCS-1), which is highly conserved from yeast to man (Stockebrand and Pongs, 2006; Burgoyne, 2007; Dason et al., 2012), has been implicated in some of these processes. Importantly, NCS-1 has been associated with psychiatric conditions including autism (Piton et al., 2008), bipolar disorder, schizophrenia (Koh et al., 2003; Bai et al., 2004), and X-linked mental retardation (Tessier and Broadie, 2011). The role, however, which NCS-1 plays in these disorders, remains unresolved in part because NCS-1 seems to have not one conserved cellular target, but multiple ones. Thus, many different target proteins have been reported as potential interaction partners of NCS-1, e.g., phosphatidylinositol 4-kinase (PI4K; Mora et al., 2002; Strahl et al., 2003), Ric8a (Mansilla et al., 2017), inositol 1,4,5-triphosphate receptor (IP_3_R; Boehmerle et al., 2006), A-type potassium channels (Nakamura et al., 2001; Guo et al., 2002), protein interacting with C kinase-1 (PICK1; Jo et al., 2008), P/Q-type Ca^2+^ channel Cav2.1 (Tsujimoto et al., 2002), D2 dopamine receptor (D2R; Kabbani et al., 2002), adenosine A_2A_ receptors (Navarro et al., 2012), and G-protein-coupled receptor kinase 1 (GRK1; Pandalaneni et al., 2015). Most of these interactions were studied mainly in cellular *in vitro* systems overexpressing NCS-1 and the physiological significance remains unclear.

In order to investigate the potential physiological roles of NCS-1, we used an NCS-1 knock-out (KO) mouse line (NCS-1^−/−^; Hermainski, 2012; Ng et al., 2016). Previous studies showed that young NCS-1^−/−^ mice are viable and generally healthy. Though NCS-1^−/−^ mice have a mild cardiac problem at a neonatal stage, i.e., a diminished systolic function, this contraction problem disappears in adulthood (Nakamura et al., 2011). NCS-1^−/−^ mice show little change in their physical activities, as determined *via* treadmill-analysis (Nakamura et al., 2017) and open field locomotion (Hermainski, 2012; Ng et al., 2016), but some behavioral deficits are notable. NCS-1^−/−^ mice showed impaired spatial learning and memory function in the Morris Water Maze Test (Nakamura et al., 2017) and a decreased willingness to work for food (Ng et al., 2016). These behavioral phenotypes were associated with a reduced release of dopamine and brain-derived neurotrophic factor (BDNF) in CA1 presynaptic neurons (Nakamura et al., 2017) and decreased presynaptic dopamine release in striatal neurons, respectively (Ng et al., 2016). Interestingly, NCS-1-deficiency also resulted in anxiety- and depressive-like behaviors as demonstrated by elevated plus maze, large open field, forced swim and tail suspension tasks (De Rezende et al., 2014).

Though behavioral phenotypes of NCS-1^−/−^ mice have been investigated extensively, their obesity, which represents the most apparent adult NCS-1^−/−^ phenotype, remained uncharacterized so far. Here we show that adult NCS-1^−/−^ mice, especially when fed a high-fat diet (HFD), are hyperglycemic and hyperinsulinemic, typical symptoms associated with obesity (Modan et al., 1985; Mehran et al., 2012). HFD-fed NCS-1^−/−^ mice display a significant increase in fat body mass. NCS-1^−/−^ adipocytes are dysfunctional giving rise to lower serum concentrations of resistin and adipokinine than adipocytes of wild-type (WT) littermates. Importantly, insulin receptor (IR) concentration in NCS-1^−/−^ adipocyte membrane is severely reduced. Apparently, NCS-1 is required directly to up-regulate IR density in adipocyte membrane ensuring an adequate insulin response to changes in diet. Our data indicate that NCS-1 plays an important role in adipocyte function and that NCS-1 deficiency yields obesity and diabetes type 2 in adult NCS-1^−/−^ mice. Combining these data with the previously reported anxiety- and depressive-like behaviors of NCS-1^−/−^ mice (De Rezende et al., 2014; Ng et al., 2016) implicates NCS-1 in a relationship of diabetes type 2 and depression, frequently observed with human patients (Nouwen et al., 2010; Renn et al., 2011; Haljas et al., 2018).

## Materials and Methods

### NCS-1 Knock-In and Knock-Out Mouse Lines

Generation of NCS-1-EGFP knockin mice and NCS-1^−/−^ mice (C57BL6/J background) has been described earlier (Hermainski, 2012; Ng et al., 2016). Briefly, NCS-1-EGFP knockin mice were generated that had the last four translated exons of Ncs-1 flanked by lox-P sites as well as an Egfp modification in exon seven to generate mice expressing NCS-1-EGFP fusion protein. Knockin mice were then crossed with CMV-Cre transgenic mice (Schwenk et al., 1995), which had been backcrossed to C57BL/6 over 10 generations, producing mice with no detectable NCS-1. NCS-1^−/−^ mice were backcrossed to C57BL/6J over 10 generations and maintained on the C57BL/6J background. Genotyping was done by PCR as described (Hermainski, 2012; Ng et al., 2016). Animal protocols were in accordance with guidelines for humane treatment of animals and were reviewed and approved by the Animals Ethics Committee of the Saarland, Germany. Mice were kept at a regular 12 h day/night cycle. They had *ad libitum* access to a normal chow diet (ssniff^TM^ R/M-H Pellets, ssniff-Spezialdiäten GmbH) and water. HFD (ssniff^TM^ EF R/M D12492, ssniff-Spezialdiäten GmbH) was fed from 6 weeks of age until the termination of the experiment as indicated in figures. Throughout we used male mice for experiments. Mice were killed by cervical dislocation at the end of the study.

### Measurement of Food and Water Consumption

Food and water consumption of mice was monitored using a nutrition monitoring system (Infra-e-Motion, Hamburg). The system also records every second cage activity of the mouse. Normal chow was provided as a powder (ssniff^TM^ EF R/M-H, ssniff-Spezialdiäten GmbH). In case of HFD, the food (ssniff^TM^ EF R/M D12492, ssniff-Spezialdiäten GmbH) was pulverized with a grinder and mixed with 25% ssniff^TM^ EF R/M-H powder to make a pourable chow. After a 5-day trial, phase food and water supply were replenished, and data collection was started for a period of 4 days. Data were transmitted online to a central computer outside the animal facility.

### Blood and Serum Analysis

Blood glucose levels were determined from whole venous tail blood using a glucose monitoring system (Roche Accu Chek). Serum was obtained by collecting blood from the submandibular vein. Blood samples were then incubated at room temperature for 60 min and centrifuged at 3,000 *g* for 10 min. Resulting serum was stored at −80°C for further use.

### Glucose and Insulin Measurements in Glucose Tolerance Tests

Glucose tolerance tests were carried out in 16-week-old animals following a 16 h fast. After measuring fasted blood glucose levels, mice were injected intraperitoneally with 2 mg glucose/g body weight (20% glucose solution, Sigma-Aldrich, St. Louis, MO, USA). Blood glucose levels were determined at 30, 60, 90, and 120 min postglucose injection. Insulin concentrations were measured after a 16 h fast and 30 min post glucose injection in blood samples taken from the *vena facialis*.

### Elisa Tests for Insulin, Adipokinines and Cytokinines

Serum concentrations of hormones were determined using the following commercially available ELISA kits: Ultra Sensitive Mouse Insulin ELISA kit (Crystal Chem), Mouse Leptin ELISA Kit (Crystal Chem), Mouse Adiponectin/Acrp30 Immunoassay (R&D Systems, Minneapolis, MN, USA), Quantikine Mouse Resistin Immunoassay (R&D Systems, Minneapolis, MN, USA), Legendplex Mouse Th2 Panel for tumor necrosis factor-alpha (TNF-α) and interleukine-6 (IL-6; BioLegend^R^, San Diego, CA, USA).

### Preparation of Fat Body Lysate

Fat body (1 g per 1 mL lysis buffer) was transferred to a Potter homogenizer and homogenized on ice in 20 mM HEPES (pH 7.4), 125 mM KCl. 0.05% Tween 80, 100 nM CaCl_2_ containing protease inhibitor cocktail according to directions of the supplier (Roche). Then 1 M sucrose was added to a final concentration of 300 mM. Lysis was continued for 45 min at 4°C on a rotarod. It was centrifuged twice for 5 min at 2,000 *g* at 4°C. The supernatant was centrifuged at 50,000 *g* for 1 h at 4°C. The supernatant was saved as cytosol fraction and kept in aliquots at −80°C until further use. The membranous pellet was resuspended in 300 mM sucrose, 10 mM HEPES (pH 7.4), 10 mM Tris-HCl (pH 8.0), 0.1 mM MgCl_2_, 100 nM CaCl_2_ buffer containing 1 tablet/10 mL protease inhibitor cocktail (Roche). Final protein concentration was ≥2 mg/mL.

### Immunoprecipitation

Protein G dynabeads were washed with 50 mM Tris-HCl (pH 7.4), 150 mM KCl, 1% (v/v) Triton X-100, 100 nM CaCl_2_ buffer containing 1 tablet/10 mL protease inhibitor cocktail (Roche). Fifty microliter Dynabead suspension was added to 500 μL lysate and incubated for 3 h at 4°C for preclearing. The beads were discarded. Primary antibodies [rabbit anti-IR antibody (5 μg/mg lysate) Abcam ab137747; rabbit anti-NCS-1 antibody (1:100) Cell Signaling D12D2] were added and for control mouse immunoglobulin (5 μg/mg lysate—Invitrogen 02-6502). Then dynabeads were collected and washed repeatedly in 50 mM Tris-HCl (pH 7.4), 150 mM KCl, 0.5% (v/v) Triton X-100, 5 mM MgCl_2_, 100 nM CaCl_2_ buffer containing 1 tablet/10 mL protease inhibitor cocktail (Roche). Finally, Dynabeads were incubated with 40 μL NuPage sample buffer and heated 10 min at 70°C before loading for PAGE.

### Page Analysis of Fat Body Lysate

PAGE was carried out using the NuPAGE 4%–12% Bis-Tris Mini Gel System according to the manufacturer’s specifications (ThermoFisher, Waltham, MA, USA). Proteins were transferred onto a PVDF membrane. Immunodetection was performed using the following antibodies: rabbit anti-IR (1:1,000 Abcam ab 137747), rabbit anti-NCS-1 (1:3,000 Cell Signaling D12D2), mouse anti—β-actin (1:5,000 Sigma-Aldrich, St. Louis, MO, USA 32430), rabbit anti-IRS-1 (1:1,000 Novus biologicals, Centennial, CO, USA NB100-82001), rabbit anti-IRS-2 (1:1,000 Cell Signaling 4502S), goat anti-rabbit IgG (HRP modified; 1:5,000 Millipore AQ132P).

### Densitometry Analysis

Scanned films of Western blots were analyzed and quantified using ImageJ software available in the public domain.

### Preparation of Paraffin-Embedded Adipose Tissue Sections

Mice were perfused with Zinc-Formal-Fixx^TM^ (ThermoFisher Scientific, Waltham, MA, USA). Gonadal fat tissue was embedded in paraffin and sectioned at 8 μm. Deparaffinized and rehydrated tissue sections were stained with anti-NCS-1 or anti-GFP antibodies followed by incubation with biotinylated secondary anti-rabbit- and, respectively, anti-chicken IgG antibodies and staining with ABC/DAB solutions (Vector Laboratories).

### Immunostaining of Pancreatic Islets

Sixteen micrometer cryosections of pancreatic islets of adult NCS-1^−/−^, NCS-1-EGFP, and WT littermates were immunostained with rabbit anti-GFP (1:2,000, Invitrogen, Carlsbad, CA, USA A11122), rabbit anti-NCS-1 (1:3,000, Cell Signaling D12D2) or rabbit anti-insulin antibody (1:1,000, Abcam 63820) followed by incubation with secondary goat anti-rabbit IgG labeled with Alexa Fluor^TM^ 488 (1:1,000, Invitrogen, Carlsbad, CA, USA A-11034) or Alexa Fluor^TM^ 546 dye. Confocal images were generated with a Leica TCS SP2 confocal microscope.

### Statistical Analysis

All numerical data are given as mean ± SEM. Repeated measurements MANOVA with Bonferroni *post hoc* test or ANOVA and Bonferroni *post hoc* test were used for statistical analysis with the help of the programme *Prism5* (GraphPad). Statistical significance is indicated with * for *P* < 0.05, ** for *P* < 0.01, *** for *P* < 0.001. Replicates and number of animals are indicated in Legends.

## Results

### NCS-1^−/−^ Mice Are Obese

**Figure 1:**
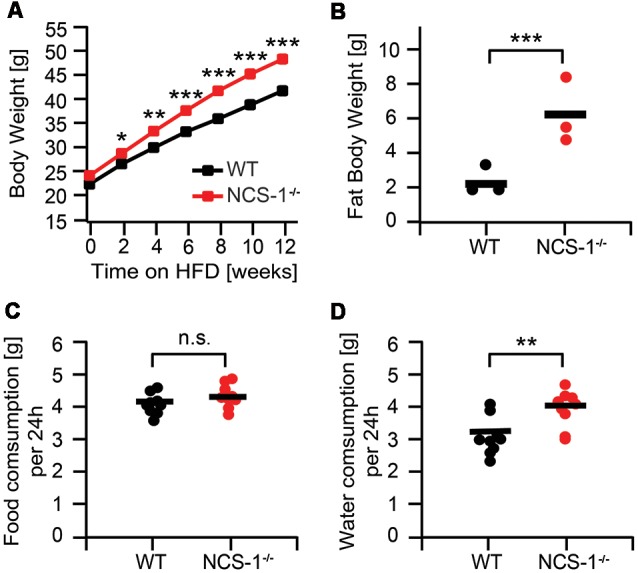
High-fat diet (HFD) induced obesity in neuronal calcium sensor-1 (NCS-1^−/−^) mice. **(A)** Body weight of male NCS-1^−/−^ mice (*n* = 29) and wild-type (WT) littermates (*n* = 29). HFD started at week 6. **(B)** Weight of fat body isolated from 30-week-old NCS-1^−/−^ mice and WT littermates. **(C)** Food consumption of NCS-1^−/−^ mice and WT littermates fed HFD. Food consumption was averaged over 24 h for a period of 4 days. Measurements were started at an age of 26 weeks. **(D)** Water consumption of NCS-1^−/−^ mice and WT littermates fed HFD. Water consumption was averaged over 24 h for a period of 4 days. Measurements were started at an age of 26 weeks. Bars represent mean values. n.s.—not significant; *P < 0.05; **P < 0.01; ***P < 0.001 (MANOVA and Bonferroni *post hoc* test).

In agreement with previous reports (Nakamura et al., 2011; Hermainski, 2012; Ng et al., 2016), we observed that NCS-1^−/−^ mice gain with onset of adulthood considerably more weight than their WT littermates (Supplementary Figure S1A). Body weights of 16-week-old NCS-1^−/−^ mice were on average ~10% higher than those of WT littermates [NCS-1^−/−^: 33.6 ± 0.7 g (*n* = 26); WT: 30.6 ± 0.6 g (*n* = 26); *P* < 0.01]. When mice were fed HFD, differences in body weight between NCS-1^−/−^ mice and WT littermates readily enlarged (Figure 1A). For example, NCS-1^−/−^ mice fed for 6 weeks HFD, had gained more weight than WT-littermates such that the NCS-1^−/−^ mice had become obese displaying a body mass index (BMI) of 36.1 ± 0.5 g/dm^2^ (*n* = 6), whereas WT-littermates were overweight, displaying a BMI of 28.5 ± 0.6 g/dm^2^ (*n* = 6; *P* = 0.002). Sixteen week-old NCS-1^−/−^ mice, having been fed HFD for 12 weeks, weighed on average ~16% more than HFD fed WT littermates [NCS-1^−/−^: 48.3 ± 0.8 g (*n* = 26); WT: 41.7 ± 0.8 g (*n* = 26); *P* < 0.001]. BMIs were 46.3 ± 0.8 g/dm^2^ (*n* = 6) and 36.4 ± 0.6 g/dm^2^ (*n* = 6) respectively. Obesity is characterized by an excess of adipose tissue (World Health Organization, 2015). Also, weight differences of HFD-fed NCS-1^−/−^ and WT mice were mainly due to a significant increase in NCS-1^−/−^ fat body mass [NCS-1^−/−^: 6.62 ± 2.2 g (*n* = 6); WT: 2.2 ± 0.47 g (*n* = 6); *P* < 0.001] (Figure 1B). Note cardiac tissue mass shows no significant difference between WT and NCS-1^−/−^ mice (Nakamura et al., 2011). Taken together, the data indicates that NCS-1^−/−^ mice, especially HFD-fed ones, are obese.

Excessive food intake and lack of physical activity combined with genetic susceptibility are the most common causes of obesity (Haslam and James, 2005). We wanted to know whether this also applies to obesity observed in NCS-1^−/−^ mice. Therefore, we investigated their feeding behavior and their general physical activity (cage activity). The respective nutrition monitoring results showed no significant difference in eating and drinking behavior between chow-fed NCS-1^−/−^ and WT mice (Supplementary Figures S1B,C). Twenty to twenty-five-week-old NCS-1^−/−^ mice daily consumed on average (*n* = 12) 3.63 ± 0.13 g chow and drank 3.69 ± 0.15 mL water. WT littermates ate and drank similar daily quantities [3.83 ± 0.16 g chow (*n* = 13); 3.37 ± 0.16 mL water (*n* = 13)]. Also, daily food consumption of HFD-fed NCS-1^−/−^ and WT mice showed no significant difference [WT: 4.05 ± 0.10 g (*n* = 9); NCS-1^−/−^: 4.29 ± 0.12 g (*n* = 9); *P* = 0.074] (Figure 1C). On the other hand, HFD-fed NCS-1^−/−^ mice apparently drank on average more than their WT littermates [NCS-1^−/−^: 3.93 ± 0.19 mL/24 h (*n* = 9); WT: 3.09 ± 0.19 (*n* = 9); *P* = 0.003] (Figure 1D). As a measure for their physical activity, we registered over a period of 96 h how many seconds per minute the mice moved in their cage. Twenty-four hours data averaged over four consecutive days (*n* = 12) showed that there was no difference in cage activity of NCS-1^−/−^ and WT mice (Supplementary Figure S2A). It corroborates our previous data that NCS-1^−/−^ mice and WT littermates show similar levels of open field locomotion (Ng et al., 2016). When fed HFD, both types of mice displayed reduced cage activity (Supplementary Figure S2B). Again, there was no significant difference and NCS-1^−/−^ and WT cage activities were alike. The results suggested that NCS-1^−/−^ obesity most likely had other reasons than excessive feeding behavior and lack of physical activity.

### NCS-1^−/−^ Mice Are Hyperglycemic and Hyperinsulinemic

**Figure 2:**
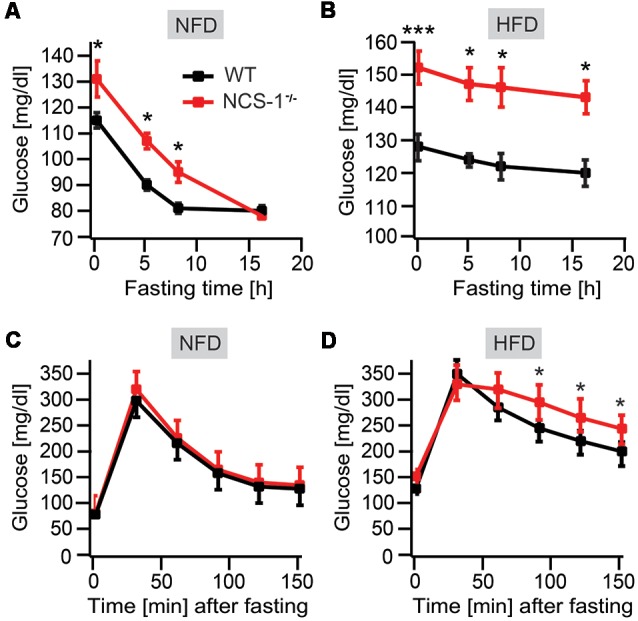
Altered glucose homeostasis in NCS-1^−/−^ mice.** (A)** Blood glucose concentration in 16-week-old NCS-1^−/−^ mice (*n* = 9) and WT littermates (*n* = 8) fed normal chow (NFD) and fasted for the times indicated. **(B)** Blood glucose concentration in 16-week-old NCS-1^−/−^ mice (*n* = 10) and WT littermates fed HFD and fasted for the times indicated. **(C)** Glucose tolerance test performed with 20-week-old NCS-1^−/−^ mice (*n* = 10) and WT littermates (*n* = 7) fed normal chow (NFD) and fasted for 16 h. **(D)** Glucose tolerance test performed with 20-week-old NCS-1^−/−^ mice (*n* = 10) and WT littermates (*n* = 7) fed HFD and fasted for 16 h. Values are presented as mean ± SEM. *P < 0.05; ***P < 0.001 (MANOVA and Bonferroni *post hoc* test).

Obesity, altered glucose homeostasis and hyperinsulinemia are closely linked (Mehran et al., 2012). Therefore, we hypothesized that obesity observed in NCS-1^−/−^ mice also was associated with hyperglycemia and hyperinsulinemia. Indeed, blood glucose levels were significantly higher in NCS-1^−/−^ mice than in WT littermates [NCS-1^−/−^: 131 ± 7 mg/dL (*n* = 8); WT: 115 ± 3 mg/dL (*n* = 9); *P* < 0.05]. Upon fasting, blood glucose levels in NCS-1^−/−^ mice and WT littermates decreased with comparable rates, reaching similar end points [WT: 80 ± 2 mg glucose/dL (*n* = 8); NCS-1^−/−^: 78 ± 2 mg glucose/dL (*n* = 9); *P* > 0.05] (Figure 2A). Evidently, NCS-1^−/−^ mice needed considerably more time for reaching the same endpoint in glucose concentration than WT littermates (NCS-1^−/−^: ~13 h; WT: 8 h), because at the onset of fasting they had a higher blood glucose concentration (Figure 2A). Furthermore, HFD produced in NCS-1^−/−^ mice a significantly higher increase in blood glucose concentration than in WT littermates resulting in concentrations of 152 ± 5 mg glucose/dL blood (*n* = 8) in NCS-1^−/−^ mice vs. 128 ± 4 mg glucose/dL blood (*n* = 10) in WT littermates (*P* < 0.001). Upon fasting, blood glucose concentrations also decreased at similar rates in HFD-fed NCS-1^−/−^ and WT mice (Figure 2B). In comparison to normal-chow fed mice, rates in HFD-fed mice were, however, nearly 10-fold slower (NCS-1^−/−^: 0.56 ± 0.02 mg glucose/dL per hour; WT: 0.50 ± 0.02 mg glucose/dL per hour). Subsequently, we measured glucose clearance after intraperitoneal injection of 2 mg glucose/g body mass into normal chow- and HFD-fed mice, which had been fasted for 16 h. Then blood glucose concentrations were analyzed at 30 min time intervals (Figures 2C,D). The results showed no significant difference in glucose clearance rates of NCS-1^−/−^ and WT mice [NCS-1^−/−^: 60.7 ± 6 mg glucose/dL per hour (*n* = 7); WT littermates: 58.6 ± 7.3 mg glucose/dL per hour (*n* = 10); *P* > 0.05] as well as in those measured between 60 and 150 min for HFD-fed NCS-1^−/−^ and WT [NCS-1^−/−^: 51 ± 15 mg glucose/dL; WT: 45 ± 12 mg glucose/dL per hour; *P* > 0.05] (Figure 2D). In conclusion, the data indicate that NCS-1 deficiency has no influence on glucose clearance rates. However, it gives rise to a significant increase in the steady-state level of blood glucose rendering NCS-1^−/−^ mice hyperglycemic.

**Figure 3:**
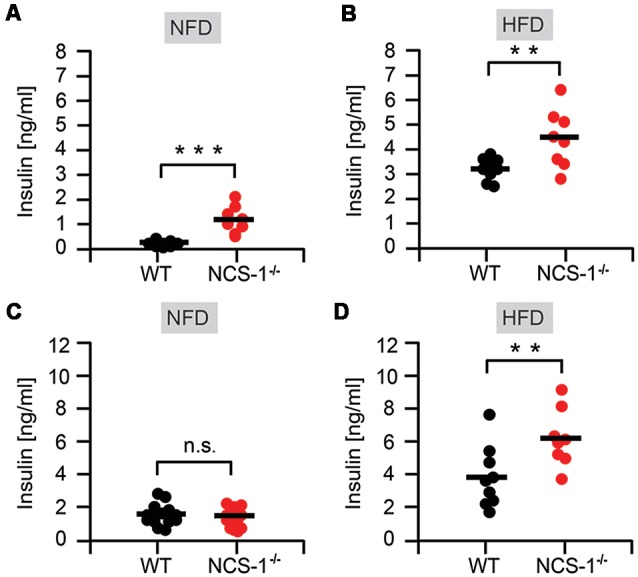
Hyperinsulinemia in NCS-1^−/−^ mice. **(A)** Plasma insulin concentration in 20-week-old WT and NCS-1^−/−^ mice fed normal chow (NFD) and fasted for 16 h. **(B)** Plasma insulin concentration in 20-week-old WT and NCS-1^−/−^ mice fed HFD and fasted for 16 h. **(C)** Plasma insulin concentration in glucose tolerance test. WT- and NCS-1^−/−^-mice fed normal chow (NFD) and fasted, were injected intraperitoneally with 2 mg glucose/g body mass. Plasma insulin concentrations were determined 30 min later. **(D)** WT- and NCS-1^−/−^-mice fed HFD and fasted, were injected intraperitoneally with 2 mg glucose/g body mass. Plasma insulin concentrations were determined 30 min later. Values are presented as mean ± SEM. n.s.—not significant; **P < 0.01; ***P < 0.001 (MANOVA and Bonferroni *post hoc* test).

Next, we tested NCS-1^−/−^ mice for hyperinsulinemia. We determined plasma insulin concentrations in NCS-1^−/−^ mice and their WT littermates, both after fasting and in response to intraperitoneal glucose injection. A 16-h fast gave rise in normal chow-fed NCS-1^−/−^ mice to a six-fold higher plasma insulin concentration than in normal chow-fed WT littermates [NCS-1^−/−^: 1.18 ± 0.19 ng insulin/mL (*n* = 8); WT: 0.20 ± 0.03 ng insulin/mL (*n* = 9);* P* < 0.001] (Figure 3A). Fasted HFD-fed mice exhibited a dramatic increase in plasma insulin concentration (Figure 3B). Again, plasma insulin concentrations were significantly higher in NCS-1^−/−^ mice [4.43 ± 0.41 ng insulin/mL (*n* = 8), *P* < 0.001] than in controls [3.23 ± 0.13 ng insulin/mL (*n* = 10), *P* < 0.01] (Figure 3B). Intraperitoneal injection of glucose (2 mg/g body mass) into fasted normal chow-fed mice significantly increased plasma insulin concentration in WT littermates from 0.20 ± 0.03 ng insulin/mL to 1.5 ± 0.16 ng insulin/mL (*n* = 15); *P* < 0.001. In contrast, glucose injection into normal chow-fed NCS-1^−/−^ mice produced an insignificant increase of the already elevated plasma insulin concentration, which was 1.18 ± 0.19 ng insulin/mL (*n* = 8) before and 1.30 ± 0.16 ng insulin/mL (*n* = 13) after intraperitoneal glucose injection (*P* = 0.31; Figures 3A,C). On the other hand, plasma insulin concentration in fasted HFD-fed WT littermates was raised to 3.81 ± 0.62 ng insulin/mL (*n* = 9; Figure 3D), whereas the one in fasted HFD-fed NCS-1^−/−^ mice had increased to an even much higher level (6.17 ± 0.61 ng insulin/mL, *n* = 8, *P* < 0.01; Figures 3B,D). Elevated levels of plasma insulin concentration in NCS-1^−/−^ mice indicates that NCS-1^−/−^ mice are hyperinsulinemic. Taken together we observed in the data that HFD-fed NCS-1^−/−^ mice are obese, hyperglycemic and hyperinsulinemic indicating a default of NCS-1^−/−^ tissue in their response to insulin.

### NCS-1 Deficiency Affects Adipokine Serum Concentration

Prime targets of insulin are muscle, liver and fat cells. NCS-1 expression is undetectable in liver and muscle (Nef et al., 1995), but prominant in adipose tissue. Western blots of fat body lysate and immunostaining of paraffin-embedded slices of gonadal adipose tissue demonstrated that both small and large adipocytes express NCS-1 (Supplementary Figures S3A,B). Since obesity-linked insulin resistance may be associated with dysfunctional adipocytes (Friedman and Haalas, 1998; Steppan et al., 2001; Galic et al., 2010; Ouchi et al., 2011), we concentrated on adipocytes for further analysis of the NCS-1^−/−^ phenotype. Adipocytes are an endocrine organ and source of adipokines and cytokines, for example, of leptin, TNF-α, IL-6, adiponectin, and resistin, which have been implicated in modulating insulin sensitivity and energy balance (Friedman and Haalas, 1998; Berg et al., 2001; Steppan et al., 2001; Mojiminiyi et al., 2007; Galic et al., 2010; Ouchi et al., 2011). Normally, plasma concentration of leptin, an important hormone involved in the regulation of energy expenditure, positively correlates with body mass (Friedman and Haalas, 1998). Also, our data revealed, a positive correlation between body mass and leptin for normal chow- and HFD-fed NCS-1^−/−^ mice and WT littermates. Furthermore, TNF-α and IL-6 plasma concentrations were just as positively correlated with NCS-1^−/−^ and WT body weight (Supplementary Figures S4A–C). It follows that plasma concentrations of leptin were positively correlated with TNF-α and IL-6 (Supplementary Figure S4D). This is in good agreement with previous data that leptin increases the production of TNF-α and IL-6 (Galic et al., 2010; Ouchi et al., 2011). Thus, deficiency of NCS-1 has no effect on the secretion of leptin, TNF-α and IL-6, respectively.

**Figure 4:**
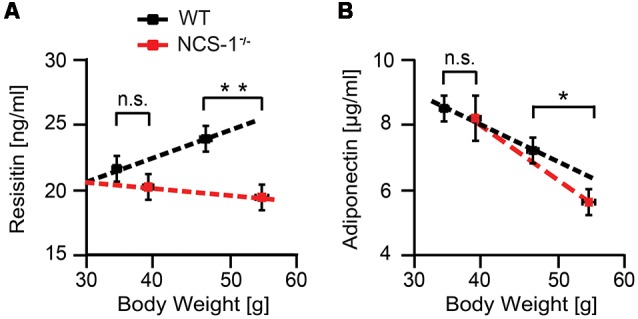
Reduced concentrations of resistin and adiponectin in NCS-1^−/−^ serum. **(A)** Relation between resistin serum concentration and body weight for 25-week-old NCS-1^−/−^ mice (*n* = 8–10) and WT littermates (*n* = 8–10). **(B)** Relation between adiponectin concentration and body weight for 25-week-old NCS-1^−/−^ mice (*n* = 8) or WT littermates (*n* = 10). Mice were kept either on normal chow (*n* = 10) or fed HFD after week 6 (*n* = 8). Bars represent mean values ± SEM. n.s.—not significant; *P < 0.05; **P < 0.01 (MANOVA and Bonferroni *post hoc* test).

In rodents, adipocytes are the primary source of resistin. It may also influence the ability of the body to respond to insulin and metabolize glucose, and it may decrease secretion of adiponectin, an insulin-sensitizing adipocytokine that seems to play an opposite role to the one of resistin (Berg et al., 2001; Steppan et al., 2001; Fasshauer et al., 2002; Möhlig et al., 2002; Mojiminiyi et al., 2007; Ouchi et al., 2011; Chen et al., 2014). Resistin levels in serum of normal chow-fed NCS-1^−/−^ and WT littermates showed no significant difference [NCS-1^−/−^: 20.2 ± 1.0 ng resistin/mL (*n* = 10); WT: 21.6 ± 0.9 ng resistin/mL (*n* = 10); *P* > 0.05] In contrast, HFD-fed NCS-1^−/−^ and WT mice displayed significantly different resistin concentrations (NCS-1^−/−^: 19.4 ± 0.7 ng resistin/mL, *n* = 8; WT: 23.9 ± 0.8 ng resistin/mL, *n* = 10, *P* = 0.008; Figure 4A). Whereas resistin serum concentrations were positively correlated with WT body mass, those in NCS-1^−/−^ mice were uncorrelated or even negatively correlated with body mass (Figure 4A). Likewise, normal chow-fed NCS-1^−/−^ mice and WT littermates showed no significant difference in adiponectin serum concentration (NCS-1^−/−^: 8.5 ± 0.4 μg adiponectin/mL, *n* = 10; WT: 8.2 ± 0.7 μg/mL, *n* = 10; *P* > 0.05; Figure 4B). In agreement with data in the literature (Berg et al., 2001; Steppan et al., 2001; Fasshauer et al., 2002; Möhlig et al., 2002; Mojiminiyi et al., 2007; Chen et al., 2014), adiponectin concentrations were negatively correlated with body mass in both types of mice (Figure 4B). But the negative correlation between body mass and adiponectin serum concentration was in HFD-fed NCS-1^−/−^ mice (*n* = 10) more pronounced than in HFD-fed WT littermates (*n* = 8). The data indicate that NCS-1 deficiency affects resistin and adiponectin plasma levels. The fact that both levels are influenced by insulin (Berg et al., 2001; Fasshauer et al., 2002; Möhlig et al., 2002; Mojiminiyi et al., 2007; Ouchi et al., 2011; Chen et al., 2014), suggests that NCS-1 deficiency affects insulin sensitivity of NCS-1^−/−^ adipocytes.

### NCS-1 Deficiency Affects Insulin-Receptor Concentration in Adipocyte Membranes

**Figure 5:**
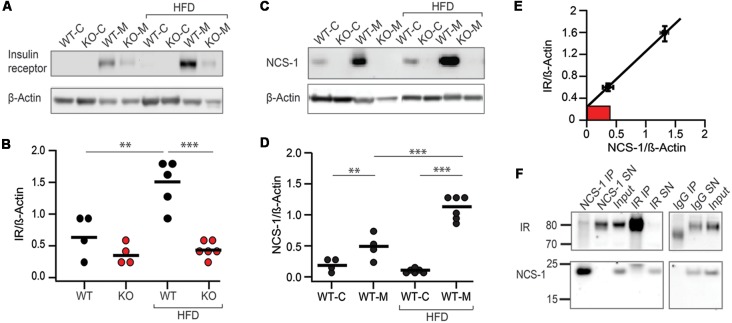
NCS-1 and insulin receptor (IR) concentration in adipocyte membrane are correlated. **(A)** Western blot analysis of cytosol (C) and membrane (M) fraction of fat body lysate prepared from NCS-1^−/−^ knockout (KO) and WT mice fed either normal chow or HFD. Blot was immunostained with anti-IR and with anti–β-actin antibodies as indicated at left. **(B)** Normalized IR signal intensities (IR/β-actin) obtained from Western blots of WT (*n* = 2–3) and NCS-1^−/−^ (KO; *n* = 2–3) fat body lysate as exemplified in **(A)**. IR concentrations (IR/β-actin) were determined in duplicate. **(C)** Western blot analysis of cytosol (C) and membrane (M) fraction of fat body lysate prepared from NCS-1^−/−^ (KO) and WT mice fed either normal chow or HFD. Blot was immunostained with anti-NCS-1 and with anti—β-actin antibodies as indicated at left. **(D)** Normalized NCS-1 signal intensities (NCS-1/β-actin) obtained from Western blots of WT fat body lysate (*n* = 2–3) as exemplified in **(C)**. NCS-1 concentrations (NCS-1/β-actin) were determined in duplicate. **(E)** Plot of normalized IR concentration vs. NCS-1 concentration. Values (± SEM) were calculated from the data shown in **(B,D)**. Red box represents IR concentration measured in lysate of NCS-1^−/−^ fat body as shown in **(B)**. **(F)** Western blot analysis of immunoprecipitates obtained after incubation of fat body lysate with anti-NCS-1 antibodies (NCS-1 IP), anti-IR antibodies (IR IP) or with non-specific immunoglubulin (IgG IP). “SN” corresponds to supernatant of the immunoprecipitation reaction and “input” to starting material. Western blots were stained either with anti-IR or with anti-NCS-1 antibody as indicated at left. Molecular weights are given in kDa at left. Bars represent mean values. **P < 0.01; ***P < 0.001 (unpaired two-tailed Student’s *t*-test).

Next, we asked if NCS-1 deficiency affects the insulin response of NCS-1^−/−^ adipocytes. Unexpectedly, we found that the adipocyte membrane of NCS-1^−/−^ mice contained significantly less IR than WT littermates. Quantitative evaluation of Western blots of membrane fractions from fat body lysate of normal chow-fed NCS-1^−/−^ and WT mice showed that IR signal intensities normalized to those of actin were about twice as high in WT than in NCS-1^−/−^ mice (IR/β-actin—WT: 0.61 ± 0.04, *n* = 4; NCS-1^−/−^: 0.30 ± 0.06, *n* = 4; *P* = 0.006; Figures 5A,B). Analysis of IR concentration in adipocyte membrane of HFD-fed WT mice revealed a significant rise that nearly tripled IR concentration (IR/β-actin: 1.60 ± 0.14, *n* = 5, *P* = 0.007). By contrast, there was no significant increase in IR concentration in adipocyte membrane of HFD-fed NCS-1^−/−^ mice (IR/β-actin: 0.43 ± 0.05, *n* = 6, *P* = 0.12; Figure 5B). The data indicates that IR concentration in the membrane of WT adipocytes is exquisitely sensitive to diet. In contrast, IR expression in NCS-1^−/−^ adipocytes is insensitive to HFD. In conclusion, NCS-1 deficiency leads to a default in diet-sensitive regulation of IR concentration in adipocyte membrane.

### Increase of IR and NCS-1 Concentrations in Adipocyte Membrane Are Correlated

Obviously, NCS-1 is involved in HFD-associated IR upregulation in adipocyte membrane. When we analyzed Western blots of fat body lysate of normal chow- and HFD-fed mice, we observed that NCS-1 expression was also HFD sensitive (Figures 5C,D). The data showed that in normal chow-fed mice two-thirds of NCS-1 (63.3 ± 6.5%, *n* = 4) and in HFD-fed mice almost all NCS-1 protein (91.2 ± 4.5%, *n* = 6) resided in the membrane fraction of fat body lysate. Importantly, fat-body membrane isolated from HFD-fed mice contained more than two-fold higher amounts of NCS-1 than those of mice fed with normal chow (HFD: NCS-1/β-actin—1.12 ± 0.07; normal chow: NCS-1/β-actin—0.48 ± 0.09; *P* < 0.001; Figures 5C,D). Thus, both the concentration of NCS-1 and IR are sensitive to HFD. Next, we constructed a plot of NCS-1 vs. IR—concentrations that were observed in fat body lysate of normal chow and resepectively, HFD-fed mice and normalized to β-actin. A linear fit to the data well described the correlation between NCS-1 and IR concentrations (Figure 5E). The fit provided two important informations. First, if the NCS-1 concentration equals zero, the fit indicated a remaining normalized IR-concentration of 0.28. A very similar IR concentration was detected in the lysate of NCS-1^−/−^ adipocytes. These data indicate that there is a basal concentration of IR in the adipocyte membrane, which is independent of NCS-1 expression. Second, there is a diet-sensitive component of IR concentration, which is correlated with HFD-sensitive up-regulation of NCS-1 concentration. It suggests that NCS-1 is an important player in the regulation of IR membrane concentration in adipocytes in response to diet changes.

It has been reported that NCS-1 associates with D2 and adenosine A_2A_ receptors (Kabbani et al., 2002; Navarro et al., 2012). We investigated the possibility that NCS-1 likewise associates with IR like. NCS-1 in membrane fractions of HFD-fed mice was precipitated with anti-NCS-1 antibodies. NCS-1 immunoprecipitates were then analyzed by immunostaining respective Western-blots with anti-IR and anti-NCS-1 antibodies. For control, we used non-specific IgG. The results showed that anti-NCS-1 antibodies immunoprecipitated NCS-1 completely and co-immunoprecipitated a significant amount of IR with it (Figure 5F). In the reverse experiment we immunoprecipitated IR and co-immunoprecipitated NCS-1 with it. In this case, anti-NCS-1 antibodies stained a band, which consistently migrated slightly more slowly in PAGE (*n* = 3) than NCS-1 of NCS-1 immuno-precipitates (Figure 5F). The altered mobility of NCS-1, which may correspond to an additional band seen in overexpression experiments (e.g., Mora et al., 2002), is difficult to understand. Our anti-NCS-1 antibodies do not stain any other protein than NCS-1 (Figure 5C, Supplementary Figure S5). Also a spillover of stained light-chain IgG is very unlikely. It may reflect a NCS-1 conformation altered by binding other ions than Ca^2+^ (Choudhary et al., 2018; Tsvetkov et al., 2018) or altered by an as yet unknown post-translational modification. In summary, the co-immunoprecipitation experiments suggest that IR and NCS-1 form a complex. Whether this reflects a direct or indirect interaction between IR and NCS-1, however, requires further experiments.

## Discussion

We have made two major observations in our characterization of the obese phenotype of NCS-1^−/−^ mice, which has a late onset and only emerges in adulthood. First, the NCS-1^−/−^ mice appear to have diabetes type 2. Second, NCS-1 deficiency impairs the up-regulation of IR in adipocyte membrane in response to HFD.

The obese phenotype of HFD-fed NCS-1^−/−^ mice, which exhibit abnormally increased fat body mass, is associated with hyperglycemia, hyperinsulinemia and dysfunctional adipocytes, in good agreement with a previous report that hyperinsulinemia and obesity are associated with dysfunctional adipocytes (Ouchi et al., 2011). We showed that NCS-1 deficiency specifically affects adipokine secretion of adipocytes. HFD-fed NCS-1^−/−^ mice have significantly lower serum levels of adiponectin and resistin than HFD-fed WT littermates, whereas serum levels of leptin, IL-6, and TNF-α were unaffected. Previous data suggest that NCS-1 has a regulatory role in dopamine and hormone secretion. Thus, NCS-1^−/−^ mice exhibit decreased presynaptic dopamine release in striatal neurons (Ng et al., 2016) and reduced release of dopamine and BDNF in CA1 presynaptic neurons, respectively (Nakamura et al., 2017). Conversely, overexpression of NCS-1 in PC12 cells increased evoked growth hormone release (McFerran et al., 1998). In this context, it is of note that NCS-1 interacts with IP_3_R in cardiac tissue and that NCS-1 overexpression enhances IP_3_R-stimulated phosphorylation of CaMKII-δ stimulated phosphorylation of CaMKII; (Nakamura et al., 2011; Nakamura and Wakabayashi, 2012). Thus, NCS-1 may affect global Ca^2+^ signals. It is likely that this effect is pertinent to the proposal that NCS-1 exerts regulatory functions in exocytosis of dense core vesicles (McFerran et al., 1998). A regulatory effect of NCS-1 on regulatory Ca^2+^-signaling may provide a common molecular basis for NCS-1 effects on hormone secretion in neurons as well as in adipocytes. Note that this does not concern insulin secretion of beta cells, which do not express NCS-1 (Supplementary Figure S5).

Adipocyte membrane of HFD-fed WT mice contained significantly more NCS-1 than WT mice fed with normal chow. In the light of reports that NCS-1 is up-regulated in the prefrontal cortex of schizophrenic and bipolar patients (Koh et al., 2003; Bai et al., 2004), it is interesting to see that a metabolic insult like HFD up-regulates NCS-1. The HFD-induced up-regulation of NCS-1 in adipocyte membrane was directly correlated with that of IR. By contrast, HFD-induced up-regulation of IR is impaired in NCS-1^−/−^ adipocytes. We conclude that NCS-1 deficiency impedes HFD-sensitive up-regulation of IR density in adipocyte membrane. Our data also indicate that adipocytes require NCS-1 for HFD-sensitive IR up-regulation. The relatively low IR concentration in NCS-1 deficient adipocyte membrane may be insufficient for a normal adipocyte response to insulin and, thus, lead to the obese phenotype in NCS-1^−/−^ mice (Soli et al., 1975). The consequences of insulin signaling in adipocytes will be discussed below.

IR activation by insulin induces cytoplasmic binding of IR substrates IRS-1 and IRS-2. They play key roles in transmitting signals from the IR to intracellular pathways. Phosphorylation on multiple sites upon activation of IR regulates IRS-1 and IRS-2 distribution between membrane and cytosol and their interaction with downstream partners. Down-regulation of IRS-1 and IRS-2 is associated with an obese phenotype (Kaburagi et al., 1997; Boucher et al., 2016; Kubota et al., 2017). Our Western-blot data indicated that IRS-1 concentrations in adipocytes of HFD-fed NCS-1^−/−^ mice are significantly reduced in comparison to WT littermates, both in cytosol and membrane fractions of fat body lysate (Supplementary Figures S6A,B). This result is in good agreement with the observation that IRS-1 is in adipocytes a major target in insulin signaling. IRS-2 concentration, on the other hand, was similar in cytosol fractions of NCS-1^−/−^ and WT fat body lysate. Only membrane fractions of NCS-1^−/−^ contained a significantly reduced IRS-2 concentration in comparison to WT (Supplementary Figures S6C,D). The data shows that NCS-1 deficiency affects down-stream of IR IRS activity and signaling, most likely as a consequence of reduced IR expression in NCS-1^−/−^ adipocyte membrane. In this context, it will be important to see whether the NCS-1 effect on IR concentration and IR signaling is specific for IR or whether NCS-1 affects also other receptors, for example, insulin growth factor-1. The activation of insulin signaling induces translocation of the glucose transporter GLUT4 to the plasma membrane. We observed that GLUT4 is expressed in NCS-1^−/−^ adipocytes at a lower level in comparison to WT (Supplementary Figures S6E,F). The data suggest that adipocytes of HFD-fed NCS-1^−/−^ mice have a lower rate of glucose-uptake than adipocytes of HFD-fed WT littermates. Previously it has been reported that overexpression of NCS-1 in 3T3L1 tissue culture cells inhibits the translocation of GLUT4 to the plasma membrane involving a phosphatidyl-4 kinase-dependent pathway (Mora et al., 2002). How NCS-1 in combination with phosphatidyl-4 kinase influences the sorting and/or translocation process of GLUT4 remained however unclear. At this stage, it is difficult to compare data obtained by overexpression of NCS-1 in tissue culture cells with ours, which were obtained with NCS-1^−/−^ adipocytes in HFD-fed adult animals.

Our data indicate that there are at least two pathways for controlling IR concentration in the adipocyte membrane; one is NCS-1 independent, the other is NCS-1 dependent. The former provides a basic IR level observed in WT and NCS-1^−/−^ mice alike. This situation provides a likely explanation for phenotypic differences observed with NCS-1^−/−^ and FIRKO mice, where IR expression in adipose tissue is completely absent (Blüher et al., 2002; Entingh-Pearsall and Kahn, 2004; Boucher et al., 2014, 2016; Kubota et al., 2017). The latter involves a direct correlation between NCS-1 and IR concentrations in adipocytes. Whether this reflects a direct association between NCS-1 and IR, as suggested by our co-immunoprecipitation results, requires further study along the lines previously shown for D2- and adenosine A_2A_ receptors (Kabbani et al., 2002; Woll et al., 2011; Navarro et al., 2012). This also includes the possibility that NCS-1 attenuates IR internalization, as was shown for D2 receptor internalization when NCS-1 is overexpressed in HEK 293 cells (Kabbani et al., 2002).

Insulin resistance, decreased plasma levels of adiponectin (hypoadiponectinemia) and decreased plasma levels of resistin are reportedly related to obesity and type 2 diabetes mellitus in patients (Way et al., 2001; Fasshauer et al., 2002; Möhlig et al., 2002; Heilbronn et al., 2004; Mojiminiyi et al., 2007; Chen et al., 2014). The observed effects of NCS-1 deficiency on IR concentration and on adipokinine secretion in combination with the observed hyperinsulinemia, hyperglycemia and obesity strongly suggest that the phenotype of NCS-1^−/−^ mice resembles type 2 diabetes. Therefore, we propose NCS-1 as a novel player in the development of type 2 diabetes. In light of the potential clinical implications, it will be important to further explore insulin responses in NCS-1^−/−^ mice.

Our study extends the phenotypic characterization of NCS-1^−/−^ mice in an important aspect. It demonstrates that NCS-1 deficiency leads to diabetes type 2 and to behavioral phenotypes reminiscent of psychiatric disorders. Thus, NCS-1^−/−^ mice display depressive-like behavior in forced swim and tail suspension tests (Friedman and Haalas, 1998), and as we previously showed, NCS-1^−/−^ mice display decreased motivation to work for food and choose the less effortful option for obtaining food (Ng et al., 2016). NCS-1^−/−^ mice, therefore, exhibit a genetic link for diabetes type 2 and mood-related behaviors. In human patients, depressive symptoms and diabetes type 2 are frequently associated (Nouwen et al., 2010; Renn et al., 2011; Haljas et al., 2018). In future studies, the generation of mouse lines with specific tissue-specific NCS-1-deficiencies may provide further insights into a potential genetic link between some psychiatric disorders and the risk of being obese.

## Data Availability

All datasets generated for this study are included in the manuscript and/or the Supplementary Files.

## Author Contributions

OP conceived the project and wrote together with OR the manuscript. OP, OR, JH and KR designed the experiments, analyzed and interpreted the data. OR carried out the Western blot analyses and the immunoprecipitations. JH constructed the knock-out mouse line and measured glucose tolerance and insulin resistance. KR measured hormone levels in mouse serum.

## Conflict of Interest Statement

The authors declare that the research was conducted in the absence of any commercial or financial relationships that could be construed as a potential conflict of interest.

## Supplementary Material

FIGURE S1 Normal food and water consumption in overweight NCS-1^−/−^ mice. **(A)** Body weight of male NCS-1^−/−^ mice (*n* = 26) and wild-type (WT) littermates (WT; *n* = 26) fed normal chow. **(B)** Food consumption of NCS-1^−/−^ mice and WT littermates fed normal chow. Food consumption was averaged over 24 h for a period of 4 days. Measurements were started at an age of 26–31 weeks. **(C)** Water consumption of NCS-1^−/−^ mice and WT littermates fed normal chow. Water consumption was averaged over 24 h for a period of 4 days. Measurements were started at an age of 26–31 weeks. **(A)** Data points represent mean values ± SEM. **P* < 0.05; ***P* < 0.01; ****P* < 0.001 (MANOVA and Bonferroni *post hoc* test). **(B,C)** Bars represent mean values. n.s.—not significant.

FIGURE S2 NCS-1^−/−^ and WT mice have similar cage activity. **(A)** Four days average of 24 h profile of cage activity of NCS-1^−/−^ mice (red trace; *n* = 10) and WT littermates (black trace; *n* = 10) fed normal chow. Measurements were started at an age of 26–31 weeks. Activity was measured every second and is plotted as number of events per minute. **(B)** Four days average of 24 h profile of cage activity of NCS-1^−/−^ mice (red trace; *n* = 9) and WT littermates (black trace; *n* = 9). Measurements were started at an age of 26–31 weeks. Mice were fed high-fat diet from an age of week 6 onwards. Activity was measured every second and is plotted as the number of events per minute. Black bar indicates light/dark cycle.

FIGURE S3 Expression of NCS-1 in adipose tissue. **(A)** Western blot analysis of fat body lysate of WT and NCS-1^−/−^ mice. Blot was immunostained with anti-NCS-1 antibodies and with anti—β-actin antibodies for input control. **(B)** Eight micrometer paraffin embedded cryosections of gonadal fat tissue of NCS-1-EGFP (KI), NCS-1^−/−^ (KO) and WT mice were immunostained either with anti—GFP antibodies (EGFP – panels in upper row) or with anti-NCS-1 antibodies (NCS-1—panels in lower row). Antigen-antibody complexes were visualized with secondary biotinylated antibodies followed by staining with 3,3′-diaminobenzidine (DAB). Black scale bar—100 μm.

FIGURE S4 Relation between leptin, TNF-α, IL-6, and body weight of WT and NCS-1^−/−^ mice. **(A)** Relation between leptin concentration and body weight for 25-week-old NCS-1^−/−^ mice or WT littermates. Mice were kept either on normal chow (*n* = 7) or fed high-fat diet after week 6 (*n* = 13). Measurements were done in duplicate. **(B)** Relation between tumor necrosis factor-alpha (TNF-α) concentration and body weight for 25-week-old NCS-1^−/−^ mice or WT littermates (WT). Mice were kept either on normal chow (*n* = 5) or fed high-fat diet after week 6 (*n* = 6). Measurements were done in duplicate. **(C)** Relation between interleukin 6 (IL-6) concentration and body weight for 26-week-old NCS-1^−/−^ mice fed normal chow (*n* = 6) and WT littermates fed normal chow (*n* = 6) or high-fat diet (*n* = 6). Measurements were done in duplicate. **(D)** Correlation of leptin concentration with TNF-α and, respectively, IL-6 plasma concentrations was based on the linear relations shown in (**A,B**; Leptin vs. TNF-α) and in (**A,C**; Leptin vs. IL-6), which were used to read out respective plasma concentrations at a given body weight. **(A–C)** Data points represent mean values ± SEM.

FIGURE S5 NCS-1 is not expressed in β-cells of pancreatic islets. Sixteen micrometer cryosections of pancreatic islets of NCS-1-EGFP (KI), NCS-1^−/−^ (KO) and WT mice. Panels in upper row: KI cryosection immunostained with anti—GFP rabbit antibodies (EGFP), KO cryosection with anti-NCS-1 rabbit antibodies (NCS-1), WT cryosection with anti-NCS-1 rabbit antibodies. KO and WT cryosections were co-stained with 4′,6-diamidin-2-phenylindol (DAPI). Panels in lower row: WT cryosection immunostained with anti-NCS-1 rabbit antibodies and, respectively, with anti—insulin mouse antibodies; ovl—overlay. Secondary antibodies were Alexa Fluor^TM^ 488—coupled goat anti-rabbit IgG and, respectively, Alexa Fluor^TM^ 546—coupled goat anti-mouse IgG. White scale bar—50 μm.

FIGURE S6 Reduced insulin receptor substrate 1 and 2 concentration in NCS-1^−/−^ membrane. **(A)** Western blot analysis of cytosol (C) and membrane (M) fraction of fat body lysate prepared from NCS-1^−/−^ (KO) and WT mice fed high-fat diet (HFD). Blot was immunostained with anti—insulin receptor substrate-1 (IRS-1) and with anti—β-actin antibodies as indicated. **(B)** Normalized insulin receptor substrate-1 signal intensities (IRS-1/β-actin) obtained from Western blots of WT (*n* = 5) and NCS-1^−/−^ (KO; *n* = 5) fat body lysate as exemplified in **(A)**. Insulin receptor concentrations in lysate were determined in duplicate. **(C)** Western blot analysis of cytosol (C) and membrane (M) fraction of fat body lysate prepared from NCS-1^−/−^ (KO) and WT mice fed high-fat diet (HFD). Blot was immunostained with anti—insulin receptor substrate-2 (IRS-2) and with anti—β-actin antibodies. **(D)** Normalized insulin receptor substrate-2 signal intensities (IRS-2/β-actin) obtained from Western blots of wild type (WT; *n* = 4) and NCS-1^−/−^ (KO; *n* = 4) fat body lysate as exemplified in **(C)**. IRS-2 lysate concentrations were determined in duplicate. **(E)** Western blot analysis of cytosol (C) and membrane (M) fraction of fat body lysate prepared from NCS-1^−/−^ (KO) and WT mice fed high-fat diet (HFD). Blot was immunostained with anti – glucose transporter (GLUT4) antibodies and with anti—β-actin antibodies as indicated. **(F)** Normalized GLUT4 signal intensities (GLUT4/β-actin) obtained from Western blots of wild type (WT; *n* = 4) and NCS-1^−/−^ (KO; *n* = 4) fat body lysate as exemplified in **(E)**. Bars represent mean values. **P* < 0.05; ***P* < 0.01 (unpaired two-tailed Student’s *t*-test).
