# Supplementary figures and images for: NCS-1 Deficiency Is Associated With Obesity and Diabetes Type 2 in Mice
Source: Front Mol Neurosci. 2019 Apr 3;12:78. doi: 10.3389/fnmol.2019.00078 (PMC6456702; doi:10.3389/fnmol.2019.00078)

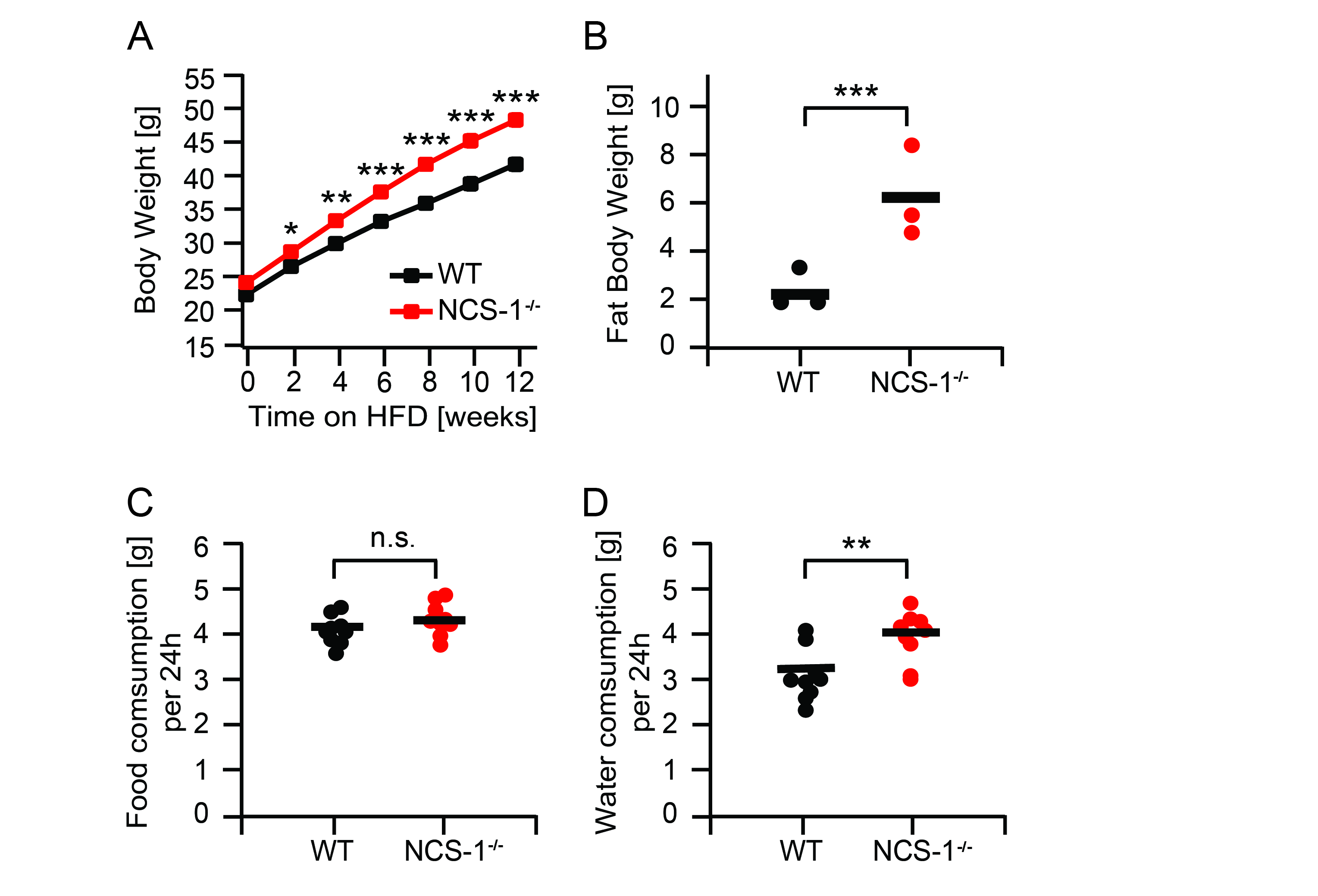

Supplement: FIGURE S1 — Normal food and water consumption in overweight NCS-1−/− mice. (A) Body weight of male NCS-1−/− mice (n = 26) and wild-type (WT) littermates (WT; n = 26) fed normal chow. (B) Food consumption of NCS-1−/− mice and WT littermates fed normal chow. Food consumption was averaged over 24 h for a period of 4 days. Measurements were started at an age of 26–31 weeks. (C) Water consumption of NCS-1−/− mice and WT littermates fed normal chow. Water consumption was averaged over 24 h for a period of 4 days. Measurements were started at an age of 26–31 weeks. (A) Data points represent mean values ± SEM. *P < 0.05; **P < 0.01; ***P < 0.001 (MANOVA and Bonferroni post hoc test). (B,C) Bars represent mean values. n.s.—not significant. [file Image_1.TIF]

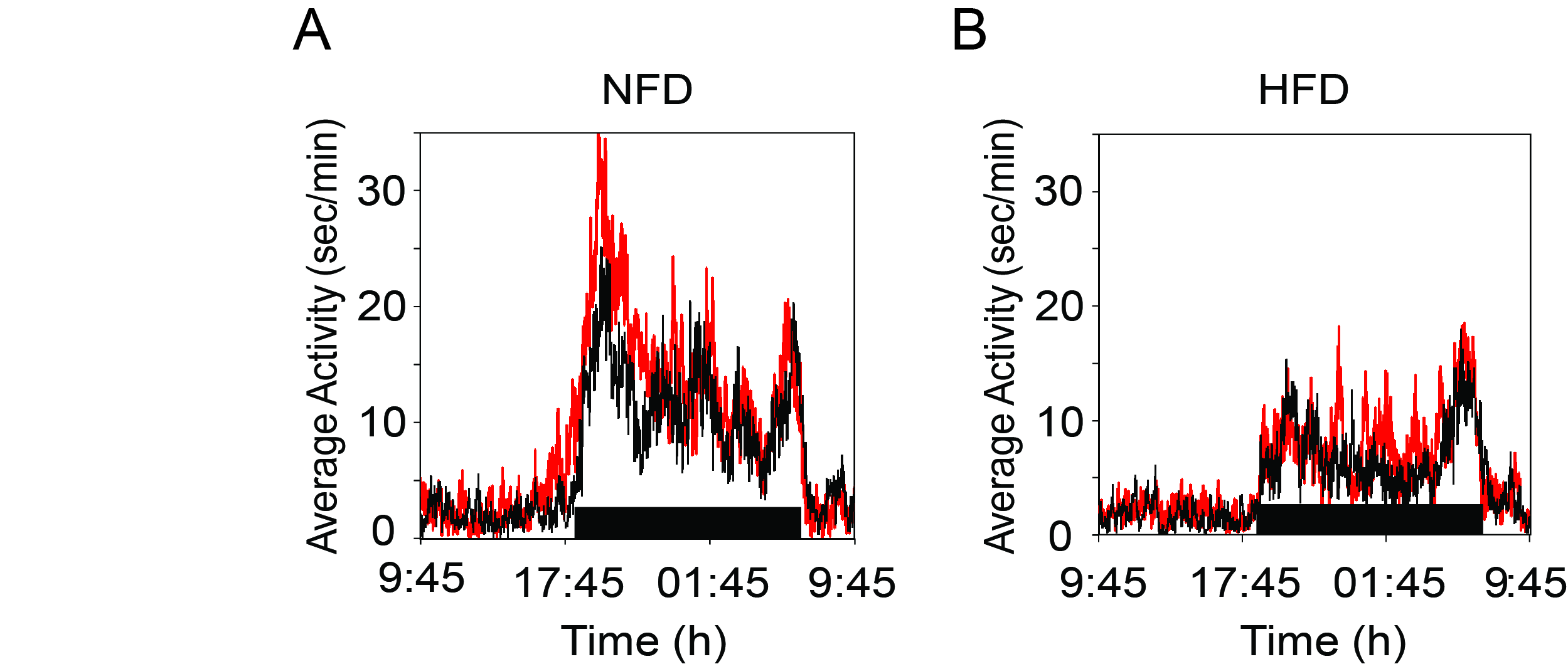

Supplement: FIGURE S2 — NCS-1−/− and WT mice have similar cage activity. (A) Four days average of 24 h profile of cage activity of NCS-1−/− mice (red trace; n = 10) and WT littermates (black trace; n = 10) fed normal chow. Measurements were started at an age of 26–31 weeks. Activity was measured every second and is plotted as number of events per minute. (B) Four days average of 24 h profile of cage activity of NCS-1−/− mice (red trace; n = 9) and WT littermates (black trace; n = 9). Measurements were started at an age of 26–31 weeks. Mice were fed high-fat diet from an age of week 6 onwards. Activity was measured every second and is plotted as the number of events per minute. Black bar indicates light/dark cycle. [file Image_2.TIF]

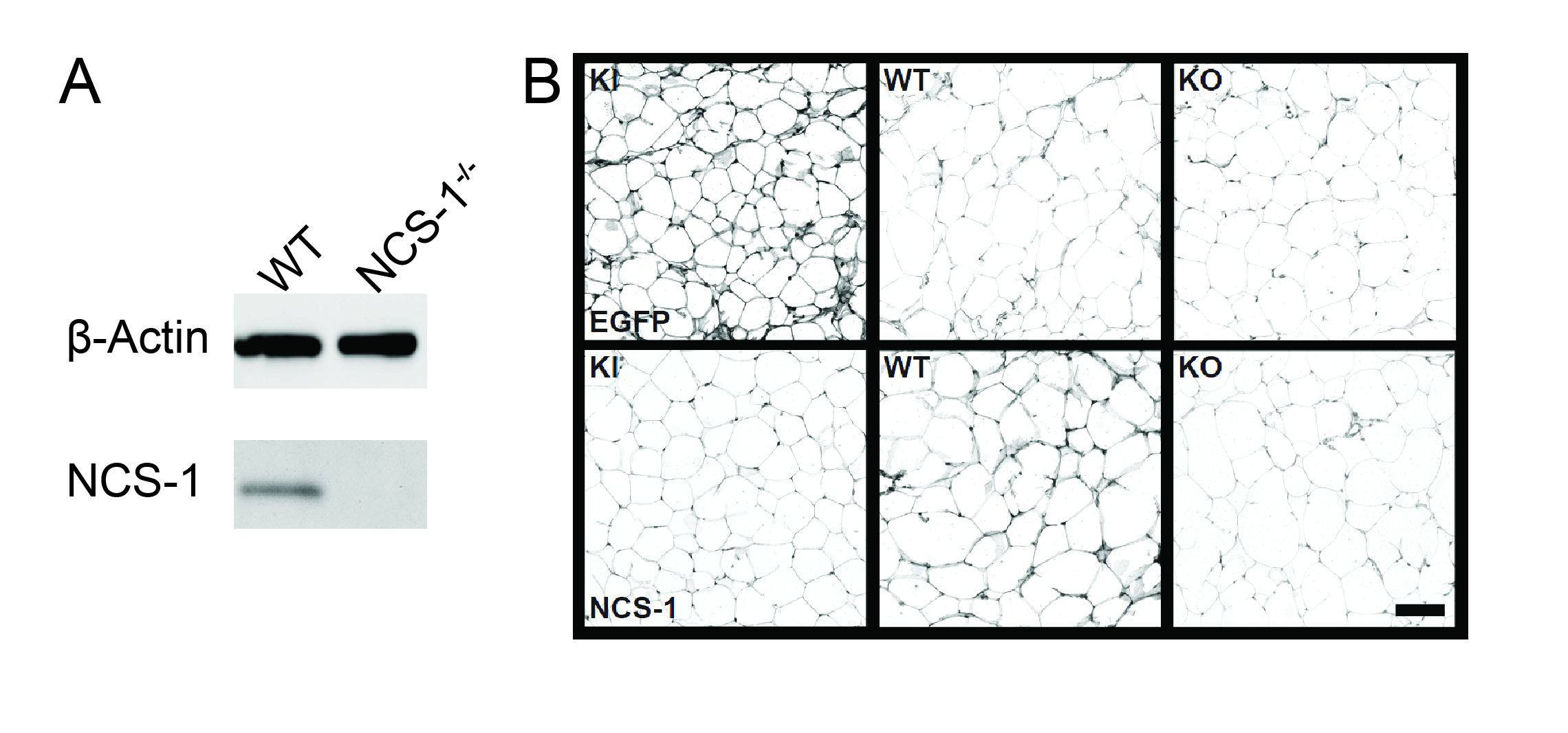

Supplement: FIGURE S3 — Expression of NCS-1 in adipose tissue. (A) Western blot analysis of fat body lysate of WT and NCS-1−/− mice. Blot was immunostained with anti-NCS-1 antibodies and with anti—β-actin antibodies for input control. (B) Eight micrometer paraffin embedded cryosections of gonadal fat tissue of NCS-1-EGFP (KI), NCS-1−/− (KO) and WT mice were immunostained either with anti—GFP antibodies (EGFP – panels in upper row) or with anti-NCS-1 antibodies (NCS-1—panels in lower row). Antigen-antibody complexes were visualized with secondary biotinylated antibodies followed by staining with 3,3′-diaminobenzidine (DAB). Black scale bar—100 μm. [file Image_3.TIF]

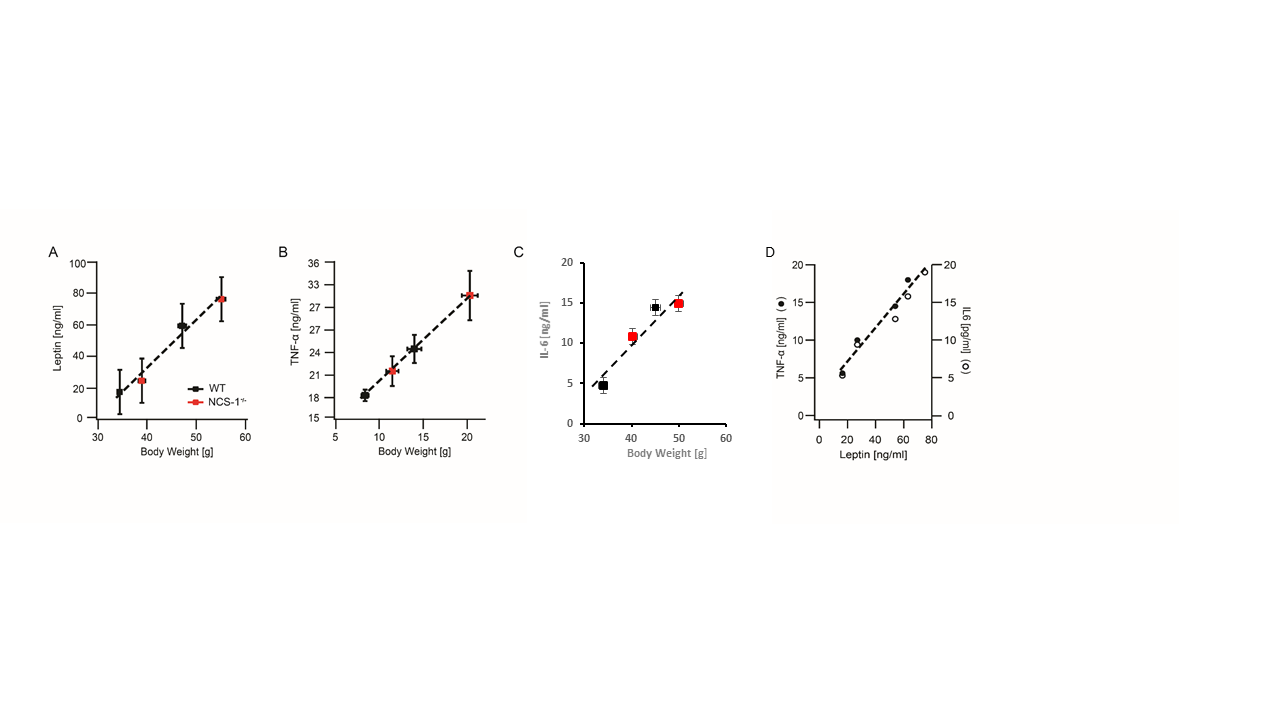

Supplement: FIGURE S4 — Relation between leptin, TNF-α, IL-6, and body weight of WT and NCS-1−/− mice. (A) Relation between leptin concentration and body weight for 25-week-old NCS-1−/− mice or WT littermates. Mice were kept either on normal chow (n = 7) or fed high-fat diet after week 6 (n = 13). Measurements were done in duplicate. (B) Relation between tumor necrosis factor-alpha (TNF-α) concentration and body weight for 25-week-old NCS-1−/− mice or WT littermates (WT). Mice were kept either on normal chow (n = 5) or fed high-fat diet after week 6 (n = 6). Measurements were done in duplicate. (C) Relation between interleukin 6 (IL-6) concentration and body weight for 26-week-old NCS-1−/− mice fed normal chow (n = 6) and WT littermates fed normal chow (n = 6) or high-fat diet (n = 6). Measurements were done in duplicate. (D) Correlation of leptin concentration with TNF-α and, respectively, IL-6 plasma concentrations was based on the linear relations shown in (A,B; Leptin vs. TNF-α) and in (A,C; Leptin vs. IL-6), which were used to read out respective plasma concentrations at a given body weight. (A–C) Data points represent mean values ± SEM. [file Image_4.tif]

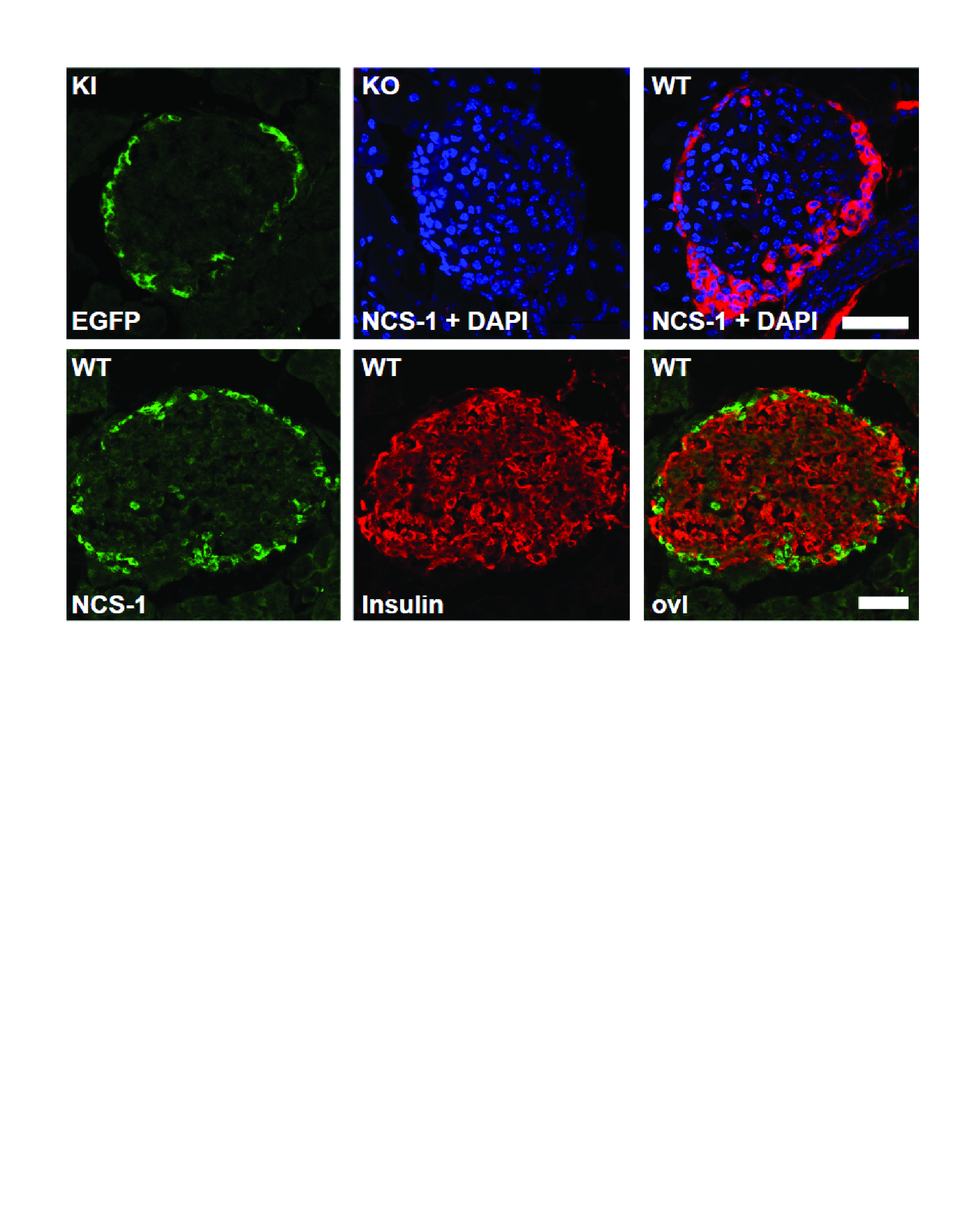

Supplement: FIGURE S5 — NCS-1 is not expressed in β-cells of pancreatic islets. Sixteen micrometer cryosections of pancreatic islets of NCS-1-EGFP (KI), NCS-1−/− (KO) and WT mice. Panels in upper row: KI cryosection immunostained with anti—GFP rabbit antibodies (EGFP), KO cryosection with anti-NCS-1 rabbit antibodies (NCS-1), WT cryosection with anti-NCS-1 rabbit antibodies. KO and WT cryosections were co-stained with 4′,6-diamidin-2-phenylindol (DAPI). Panels in lower row: WT cryosection immunostained with anti-NCS-1 rabbit antibodies and, respectively, with anti—insulin mouse antibodies; ovl—overlay. Secondary antibodies were Alexa FluorTM 488—coupled goat anti-rabbit IgG and, respectively, Alexa FluorTM 546—coupled goat anti-mouse IgG. White scale bar—50 μm. [file Image_5.TIF]

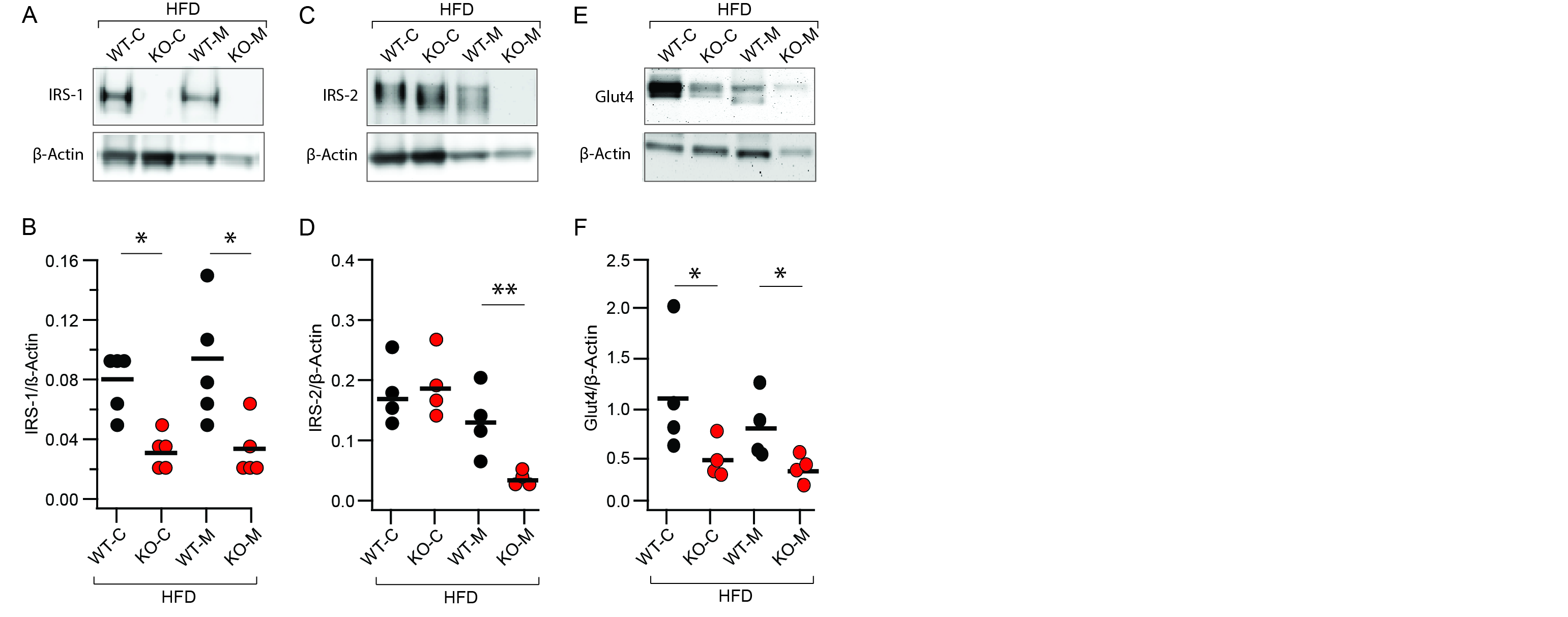

Supplement: FIGURE S6 — Reduced insulin receptor substrate 1 and 2 concentration in NCS-1−/− membrane. (A) Western blot analysis of cytosol (C) and membrane (M) fraction of fat body lysate prepared from NCS-1−/− (KO) and WT mice fed high-fat diet (HFD). Blot was immunostained with anti—insulin receptor substrate-1 (IRS-1) and with anti—β-actin antibodies as indicated. (B) Normalized insulin receptor substrate-1 signal intensities (IRS-1/β-actin) obtained from Western blots of WT (n = 5) and NCS-1−/− (KO; n = 5) fat body lysate as exemplified in (A). Insulin receptor concentrations in lysate were determined in duplicate. (C) Western blot analysis of cytosol (C) and membrane (M) fraction of fat body lysate prepared from NCS-1−/− (KO) and WT mice fed high-fat diet (HFD). Blot was immunostained with anti—insulin receptor substrate-2 (IRS-2) and with anti—β-actin antibodies. (D) Normalized insulin receptor substrate-2 signal intensities (IRS-2/β-actin) obtained from Western blots of wild type (WT; n = 4) and NCS-1−/− (KO; n = 4) fat body lysate as exemplified in (C). IRS-2 lysate concentrations were determined in duplicate. (E) Western blot analysis of cytosol (C) and membrane (M) fraction of fat body lysate prepared from NCS-1−/− (KO) and WT mice fed high-fat diet (HFD). Blot was immunostained with anti – glucose transporter (GLUT4) antibodies and with anti—β-actin antibodies as indicated. (F) Normalized GLUT4 signal intensities (GLUT4/β-actin) obtained from Western blots of wild type (WT; n = 4) and NCS-1−/− (KO; n = 4) fat body lysate as exemplified in (E). Bars represent mean values. *P < 0.05; **P < 0.01 (unpaired two-tailed Student’s t-test). [file Image_6.TIF]
